# Diethylcarbamazine activity against *Brugia malayi *microfilariae is dependent on inducible nitric-oxide synthase and the cyclooxygenase pathway

**DOI:** 10.1186/1475-2883-4-4

**Published:** 2005-06-02

**Authors:** Helen F McGarry, Leigh D Plant, Mark J Taylor

**Affiliations:** 1 Filariasis Research Laboratory, Molecular and Biochemical Parasitology, Liverpool School of Tropical Medicine, Pembroke Place, Liverpool L3 5QA, UK

## Abstract

**Background:**

Diethylcarbamazine (DEC) has been used for many years in the treatment of human lymphatic filariasis. Its mode of action is not well understood, but it is known to interact with the arachidonic acid pathway. Here we have investigated the contribution of the nitric oxide and cyclooxygenase (COX) pathways to the activity of DEC against *B. malayi *microfilariae in mice.

**Methods:**

*B. malayi *microfilariae were injected intravenously into mice and parasitaemia was measured 24 hours later. DEC was then administered to BALB/c mice with and without pre-treatment with indomethacin or dexamethasone and the parasitaemia monitored. To investigate a role for inducible nitric oxide in DEC's activity, DEC and ivermectin were administered to microfilaraemic iNOS^-/- ^mice and their background strain (129/SV). Western blot analysis was used to determine any effect of DEC on the production of COX and inducible nitric-oxide synthase (iNOS) proteins.

**Results:**

DEC administered alone to BALB/c mice resulted in a rapid and profound reduction in circulating microfilariae within five minutes of treatment. Microfilarial levels began to recover after 24 hours and returned to near pre-treatment levels two weeks later, suggesting that the sequestration of microfilariae occurs independently of parasite killing. Pre-treatment of animals with dexamethasone or indomethacin reduced DEC's efficacy by almost 90% or 56%, respectively, supporting a role for the arachidonic acid and cyclooxygenase pathways *in vivo*. Furthermore, experiments showed that treatment with DEC results in a reduction in the amount of COX-1 protein in peritoneal exudate cells. Additionally, in iNOS^-/- ^mice infected with *B. malayi *microfilariae, DEC showed no activity, whereas the efficacy of another antifilarial drug, ivermectin, was unaffected.

**Conclusion:**

These results confirm the important role of the arachidonic acid metabolic pathway in DEC's mechanism of action *in vivo *and show that in addition to its effects on the 5-lipoxygenase pathway, it targets the cyclooxygenase pathway and COX-1. Moreover, we show for the first time that inducible nitric oxide is essential for the rapid sequestration of microfilariae by DEC.

## Background

Diethylcarbamazine citrate (DEC) has been used in the treatment and control of lymphatic filariasis (caused by the nematodes *Wuchereria bancrofti*, *Brugia malayi *and *B. timori*) since 1947 and it continues to play an important role, being one of the drugs used in the Global Programme for the Elimination of Lymphatic Filariasis [1]. However, despite this long period of use, DEC's mode of action is still poorly understood. Particularly intriguing is the marked contrast between its rapid action *in vivo *and the lack of significant activity *in vitro*. *In vivo*, the response is rapid: within a few minutes of treatment, peripheral blood microfilariae counts drop dramatically [2]. The poor *in vitro *activity indicates that DEC probably requires some host factor for its activity, and previous work has highlighted the role of the innate immune system and leucocytes independent of T cells and complement in the activity of DEC [3,4].

DEC also has anti-inflammatory properties, as a result of its interference with arachidonic acid metabolism [4]. The products of the arachidonic acid metabolic pathway, eicosanoids, have a number of biological effects, including inhibition of platelet aggregation; regulation of leucocyte activation and adherence; mediation of granulocyte chemotaxis and degranulation; and promotion of vasodilatation [5]. It is well known that DEC inhibits enzymes of the 5-lipoxygenase pathway, leukotriene synthases [6,7]. Additionally, *in vitro*, DEC blocks endothelial cell production of the cyclooxygenase (COX) pathway products prostaglandin (PG) E_2_, prostacyclin (PGI_2_) and thromboxane A_2 _but has no effect on platelet prostanoid production [8]. In addition, the drug increases the rate and degree of microfilariae adherence to granulocytes, with eosinophil adhesion in particular being augmented [9-11]. Nevertheless, a role for some of these activities has yet to be demonstrated *in vivo *and so we have used a mouse model to identify the host factors responsible for the rapid efficacy of DEC.

The arachidonic acid pathway includes lipoxygenase and cyclooxygenase enzymes. The COX pathway has similarities with the nitric oxide (NO) pathway, since both have constitutive and inducible isoforms of their enzymes and are key regulators of inflammatory responses [12,13]. The COX and NO pathways are known to interact with each other, with there being 'cross-talk' between NO/PGE_2 _and iNOS/COX which is generally stimulatory but may also be inhibitory [14,15]. Therefore, we have used a combination of pharmacological inhibitors and gene-knockout technology to elucidate the role of these two pathways in DEC's activity *in vivo*.

## Materials and methods

### Parasites and mice

Microfilariae of *Brugia malayi *were obtained from TRS Laboratories (Georgia, USA), suspended in RPMI 1640 with 5% FCS, and 300000 parasites in a volume of 200 μl were injected intravenously into mice. Systemic parasitaemia was allowed to equilibrate for 24 hours, then heparinised blood samples were taken by tail bleeding and parasitaemia was measured. Mice were allocated into age- and size-matched groups and treated as described below. All animals were kept in the Biological Services Unit of the University of Liverpool in accordance with Home Office regulations and were fed and watered *ad libitum*. BALB/c mice were kept under standard conditions, and the 129/SV and targeted knockout of the iNOS gene (iNOS^-/-^, kindly provided by Prof. F.Y. Liew, University of Glasgow) strains in filter-top cages.

### Action of DEC against microfilariae *in vivo *in mice

Three BALB/c mice infected with *B. malayi *microfilariae were treated with a single, oral dose of DEC 100 mg/kg [3] (Sigma, U.K) in distilled water and the parasitaemia monitored from five minutes to two weeks post treatment. To investigate the role of the arachidonic acid metabolic pathway in the mode of action of DEC, indomethacin (10 mg/kg in 1% ethanol), water-soluble dexamethasone (3 mg/kg in water, both obtained from Sigma, U.K.), or vehicle was given by intra-peritoneal (i.p.) injection to microfilaraemic male BALB/c mice 30 minutes before oral DEC administration (100 mg/kg, three mice per treatment group). One animal was kept as an untreated control. Heparinised blood samples were taken at intervals post treatment for measurement of parasitaemia. Experiments were repeated three times.

The requirement for inducible NO in DEC's efficacy was determined in iNOS^-/- ^mice. DEC (100 mg/kg) or vehicle were administered orally to three female iNOS^-/- ^mice or their background strain, 129/SV. Mice were tail-bled at regular intervals post-treatment for evaluation of parasitaemia. To test the efficacy of another anti-filarial drug, ivermectin, in these mice, ivermectin phosphate (1 mg/kg in 1% DMSO) was administered by i.p. injection. This experiment was repeated three times.

### Expression of COX-1, COX-2 and iNOS in DEC-exposed peritoneal exudate cells

Male 129/SV and iNOS^-/- ^mice were injected i.p. with 10 mg/kg DEC in endotoxin-free water or 100 μl of endotoxin-free water (three mice in each group). After 30 minutes, peritoneal exudate cells were collected in sterile PBS with 1 g/L glucose, 1% bovine serum albumin and 1 U/ml heparin. The cells were pelleted and lysed in 1 ml TRI reagent (Sigma, U.K.) then protein extracted according to the supplied protocol.

For Western blot analysis, 10 μg of each protein were separated on a 7.5 % denaturing SDS polyacrylamide gel and blotted on to 0.45 μM pore size PVDF membrane (Immobilon P, Micropore, U.K.). After blocking overnight at 4°C in block buffer (1% casein in PBS/0.1% Tween) and washing in PBS/0.1% Tween, membranes were incubated for 1 hour in rabbit anti-mouse COX-1, COX-2 or iNOS polyclonal IgG (Cayman Chemical Co., Alexis Corporation, U.K.) diluted to 1 in 5000 in block buffer. The anti-COX antibodies showed no cross-reactivity with the opposite isoform, whilst the anti-iNOS antibody showed only 5% cross-reactivity against nNOS and none against eNOS. Membranes were then washed and incubated for 1 hour in goat anti-rabbit IgG conjugated to horse radish peroxidase (Nordic, The Netherlands) diluted to between 1 in 20000 and 1 in 100000, depending on the primary antibody, followed by further washing. The electrochemiluminescent reagent SuperSignal West (Pierce Perbio, U.K.) was used to visualise the bands on X-ray films.

### Statistical analysis

Parasitaemia data were expressed as mean percentage of pretreatment microfilariae or as a percentage of untreated control microfilaraemias per 100 μl of blood and were analysed by the two-tailed Student's *t*-test. P values of < 0.05 were considered to be significant.

## Results

### Action of DEC against microfilariae *in vivo *in mice

**Figure 1:**
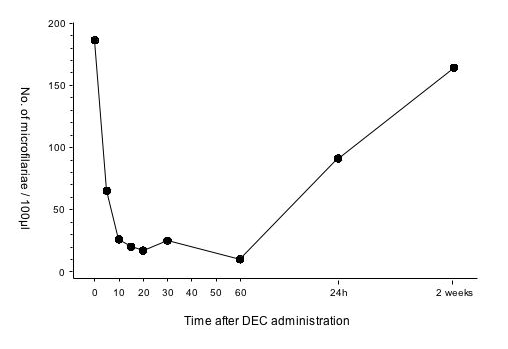
**DEC causes rapid sequestration of *B. malayi *microfilariae in BALB/c mice. **BALB/c mice intravenously injected with *B. malayi *microfilariae were dosed orally with 100 mg/kg DEC and microfilaraemia monitored from 5 to 60 minutes post treatment, then at 24 hours and two weeks.

**Figure 2:**
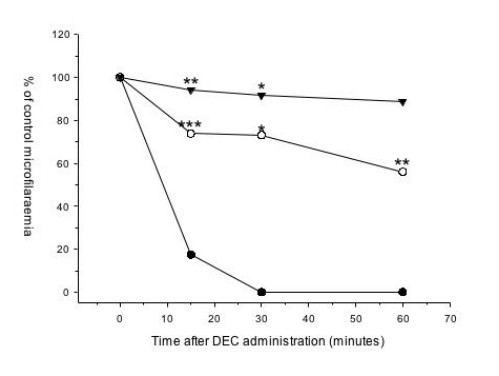
**Indomethacin or dexamethasone pre-treatment reduces efficacy of DEC in BALB/c mice infected with *B. malayi *microfilariae. **Indomethacin (10 mg/kg), dexamethasone (3 mg/kg) or vehicle was administered 30 minutes before oral dosing with DEC (100 mg/kg). Symbols are means of three mice for the DEC plus dexamethasone group (triangles), seven mice for the DEC plus indomethacin group (white circles) and four mice for the DEC-only group (black circles). Significantly different results from the DEC-only group are denoted by * (P < 0.017), ** (P = 0.001) or *** (P = 0.000).

In BALB/c mice treated with DEC alone, microfilaraemia levels were reduced by five minutes with a sustained reduction for at least 60 minutes post-treatment (Fig. 1). However, by 24 hours after treatment, microfilarial levels had partially recovered and two weeks later they had returned to levels approaching those pre-treatment (Fig. 1). Subsequent experiments focused on the rapid activity of DEC over the first one to two hours. Neither vehicle, indomethacin nor dexamethasone by itself had any effect on microfilaraemia in BALB/c mice (data not shown). However, in mice pre-treated with indomethacin or dexamethasone, microfilaraemias were reduced by only 11% (dexamethasone) or 44% (indomethacin) of untreated controls at 60 minutes post DEC administration (Fig. 2). The differences from the DEC-only group were statistically significant for all time points for indomethacin (P < 0.004) and for 15 and 30 minutes post-treatment for dexamethasone (P < 0.017) pre-treatments.

**Figure 3:**
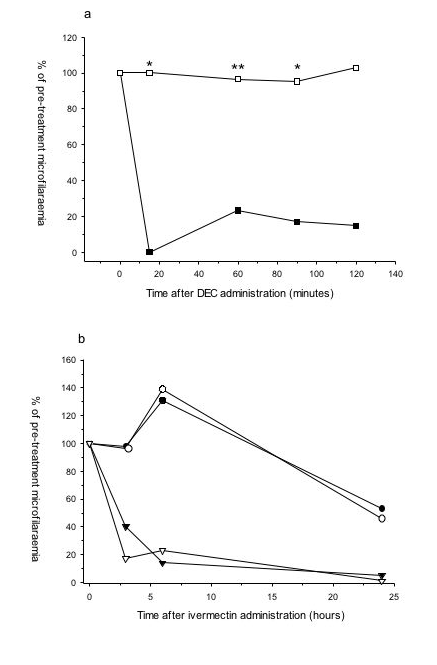
**DEC is ineffective against *B. malayi *microfilariae in the absence of iNOS. **Efficacy of (a) a single, oral dose of DEC (100 mg/kg) or (b) a single, i.p. dose of ivermectin phosphate (1 mg/kg) in 129/SV and iNOS^-/- ^mice infected with *B. malayi *microfilariae. Black symbols represent 129/SV mice, white symbols iNOS^-/-^. Squares indicate DEC administration, triangles ivermectin administration and circles untreated controls. Symbols represent mean results from at least three or four mice, except in the case of those treated with ivermectin (two animals) from two combined experiments which were representative of a further repeat. Significantly different microfilaraemias between 129/SV and iNOS^-/- ^mice after DEC administration are denoted by * (P = 0.001) or ** (P = 0.000).

DEC administration also rapidly reduced microfilaraemias in 129/SV mice but, in contrast, had no effect on microfilariae levels in iNOS^-/- ^mice, in which microfilaraemia was maintained at pre-treatment levels for at least 2 hours (Fig. 3a), with no significant differences from untreated iNOS^-/- ^controls (P > 0.887 for all time points). In contrast, ivermectin was effective in both 129/SV and iNOS^-/- ^mice (Fig. 3b), although it had a slower onset of action than DEC. However, by 24 hours no microfilariae were detected in either strain of mouse given ivermectin.

### Expression of COX-1, COX-2 and iNOS in DEC-exposed peritoneal exudate cells

**Figure 4:**
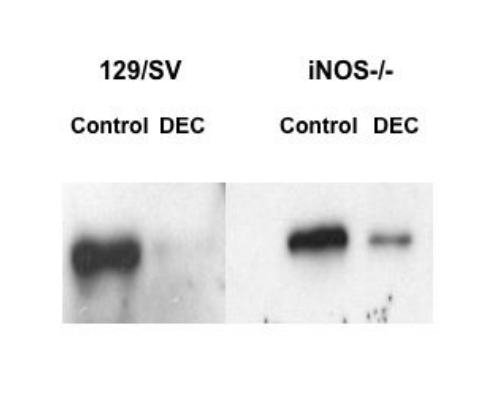
**Western blot detection of COX-1 protein from peritoneal exudate cells. **COX-1 protein was detected in 129/SV and iNOS^-/- ^peritoneal exudate cells thirty minutes after i.p. injection of endotoxin-free water (control) or DEC (10 mg/kg). Proteins (10 μg) were separated on a 7.5% denaturing SDS polyacrylamide gel, transferred to PVDF membrane, incubated with rabbit anti-mouse COX-1, then goat anti-rabbit IgG-horse radish peroxidase conjugate and detected by chemiluminescence.

Thirty minutes after administration of endotoxin-free water to 129/SV and iNOS^-/- ^mice, peritoneal exudate cells were expressing COX-1 protein, whereas those from DEC-exposed animals contained markedly less COX-1 (Fig. 4). Interestingly, there seemed to be a higher level of COX-1 remaining in the iNOS^-/- ^than the 129/SV macrophages after DEC treatment. Neither COX-2 nor iNOS protein was detected in any of the 129/SV or iNOS^-/- ^groups (not shown).

## Discussion

Here we have used a murine model to elucidate the processes within the mammalian host that contribute to DEC's rapid *in vivo *action. The involvement of two interacting pathways, the cyclooxygenase and inducible nitric oxide pathways, were shown to mediate the activity of DEC *in vivo*.

Treatment of mice with DEC resulted in a rapid reduction in microfilaraemia. This reduction, however, was transient and microfilaraemia began to recover 24 hours after treatment, with almost full restoration to pre-treatment levels two weeks after treatment. This has been previously observed in other models [16,17] and suggests that the disappearance of the microfilariae from the peripheral circulation and their sequestration in the central vascular system occur independently of parasite killing. A prolonged course of DEC treatment of *B. malayi-*infected mice led to sustained reductions in circulating microfilariae for at least 30 days [18].

Our results confirm previous findings showing that an important target for DEC is the arachidonic acid metabolic pathway. Inhibition at the first stage in the pathway by dexamethasone, which inhibits phospholipase A2, almost completely abolished the activity of DEC, whereas inhibition of the cyclooxygenase enzymes COX-1 and COX-2 by indomethacin reduced its efficacy by 56%, indicating that in addition to its well documented inhibition of the 5-lipoxygenase pathway [6,7], DEC acts on the cyclooxygenase pathway. We have shown that at least one way it does this *in vivo *is by the loss of COX-1 protein within 30 minutes of administration.

The lack of activity of DEC in mice deficient in iNOS identifies a novel enzyme system involved in the *in vivo *activity of DEC. Previously we have shown that *B. malayi *microfilariae are susceptible to nitric oxide *in vitro *[19]. However, we found no evidence that DEC itself up-regulated iNOS activity either *in vitro *(not shown) or *in vivo*, in agreement with Rajan et al. [20], who did not find any induction of NO release from murine macrophages or rat endothelial cells treated with DEC. It therefore seems probable that iNOS exerts an effect on DEC activity via its interaction with cyclooxygenases, an idea supported by the reduced loss of COX-1 protein in peritoneal exudate cells derived from iNOS^-/- ^mice. Several studies have shown that NO and iNOS interact with COX enzymes to cause an increase in enzymatic activity [21] and consequently increased prostaglandin synthesis [22-25], although large amounts of endogenous NO inhibited COX expression and activity in murine macrophages [26]. One explanation of the differential effects of NO on COX activity may relate to effects on different COX isoforms. For example, NO can activate COX-1 in fibroblasts but inhibit COX-2 in the same cell [15]. Although our studies do not distinguish between the role of COX-1 and COX-2 in DEC's activity, the rapid activity of DEC sequestration and the depletion of COX-1 protein suggest a role for COX-1. COX-1 but not COX-2 is essential for the early production of prostaglandins from macrophages and mast cells [27,28]. Further studies on mice deficient in COX isoforms or the use of isoform-specific pharmacological inhibitors could address this question. Several polymorphisms in the human iNOS gene have been described that are associated with a variety of diseases, including malaria [29-31] and hypertension [32]; it would be interesting to know if these or other polymorphisms affected responsiveness to DEC therapy.

Our findings could help expand our understanding of the mechanisms involved in the cellular processes leading to sequestration and the subsequent killing of parasites. In addition to the elevation of granulocyte adherence, platelets have also been shown to bind to and kill microfilariae [33]. In view of the well know effects of NO and prostaglandins on platelet function and evidence to suggest the presence of inducible NO in human platelets [34,35], the role of platelets in parasite sequestration and killing should be re-evaluated *in vivo*.

Filarial parasites also produce and release prostanoids, including PGE_2_, PGI_2 _and PGD_2 _[36-41], which result in inhibition of platelet aggregation [40], vasodilatation of the blood vessels and immune suppression, and may contribute to the long persistence of these parasites in their natural hosts [41]. This prostanoid production is also inhibited by DEC [8]. Significantly, they do not produce thromboxane A_2 _[36]. In contrast to mammalian systems, in which eicosanoid formation is often in response to agonist-induced stimulation, microfilariae produce prostanoids constitutively [36], but the mechanisms by which they do so have not yet been described in detail, although a glutathione *S*-transferase of *O. volvulus *synthesizes PGD_2 _from PGH_2 _[39]. It is not clear if DEC acts predominantly against the prostanoids of the worm or of the host. The lack of any direct effect of dexamethasone and indomethacin on microfilaraemia suggests that these drugs either do not influence parasite prostaglandins *in vivo *or that if they do, they are not involved in DEC-mediated sequestration. Further studies that involve inhibition of the key parasite enzymes would be required to determine the role of parasite-derived prostanoids in DEC activity. Recent studies have reported a direct activity of DEC against *Wuchereria bancrofti *microfilariae that results in exsheathment, organelle damage and cytolysis [42], which occur both *in vitro *and *in vivo *and suggest that DEC may have a direct effect on worms in addition to its interaction with host-derived pathways as reported here.

Much remains to be discovered of the mode of action of DEC. What mechanisms lead to parasite killing following sequestration in the central vasculature; and how does this relate to the paradoxical appearance of microfilariae in the peripheral circulation following the 'DEC provocative test'? What is the role of host immunity and effects on adult worms in the long-term efficacy of DEC? This model should be a powerful tool to address these questions and others to further unravel the mysteries of this elusive drug.

## Conclusion

Inducible nitric oxide synthase and the cyclooxygenase pathway were found to be essential for DEC's activity *in vivo*. Along with its well-documented activity on the lipoxygenase pathway, DEC administered *in vivo *reduced the amount of the host's COX-1. Further elucidation of DEC's mechanism of action with this murine model could provide a clearer understanding of the interaction of the nitric oxide and cyclooxygenase pathways and the cellular and molecular events at the site of sequestration.

## List of abbreviations

DEC, diethylcarbamazine citrate; COX, cyclooxygenase; i.p., intra-peritoneal; PG, prostaglandin; PGI_2_, prostacyclin; NO, nitric oxide; iNOS, inducible nitric-oxide synthase.

## Competing interests

The author(s) declare that they have no competing interests.

## Authors' contributions

HFM assisted with the *in vivo *experiments, performed the Western blot detection, analysed and interpreted the results, conducted statistical analysis and wrote the manuscript. LDP collected the parasitaemia data and assisted with the *in vivo *experiments. MJT conceived the study, performed the *in vivo *experiments, interpreted the results and advised on the manuscript. All authors read and approved the final manuscript.

## References

[B1] Molyneux DH, Bradley M, Hoerauf A, Kyelem D, Taylor MJ (2003). Mass drug treatment for lymphatic filariasis and onchocerciasis. Trends Parasitol.

[B2] Hawking F, Laurie W (1949). Action of hetrazan on filariasis and onchocerciasis. Lancet.

[B3] Vickery AC, Nayar JK, Tamplin ML (1985). Diethylcarbamazine-mediated clearance of *Brugia pahangi *microfilariae in immunodeficient nude mice. Am J Trop Med Hyg.

[B4] Maizels RM, Denham DA (1992). Diethylcarbamazine (DEC): immunopharmacological interactions of an anti-filarial drug. Parasitol.

[B5] Needleman P, Turk J, Jakschik BA, Morrison AR, Lefkowith JB (1986). Arachidonic acid metabolism. Ann Rev Biochem.

[B6] Bach MK, Brashler JR (1986). Inhibition of the leukotriene synthetase of rat basophil leukemia cells by diethylcarbamazine, and synergism between diethylcarbamazine and piriprost, a 5-lipoxygenase inhibitor. Biochem Pharmacol.

[B7] Mathews WR, Murphy RC (1982). Inhibition of leukotriene biosynthesis in mastocytoma cells by diethylcarbamazine. Biochem Pharmacol.

[B8] Kanesa-Thasan N, Douglas JG, Kazura JW (1991). Diethylcarbamazine inhibits endothelial and microfilarial prostanoid metabolism *in vitro*. Mol Biochem Parasitol.

[B9] King CH, Greene BM, Spagnuolo PJ (1983). Diethylcarbamazine citrate, an antifilarial drug, stimulates human granulocyte adherence. Antimicr Ag Chemo.

[B10] Rácz P, Tenner-Rácz K, Büttner DW, Albiez EJ (1982). Ultrastructural evidence for eosinophil-parasite adherence (EPA) reaction in human onchocercal lymphadenitis in the early period following diethylcarbamazine treatment. Tropenmed Parasitol.

[B11] Medina-De la Garza CE, Brattig NW, Tischendorf FW, Jarrett JMB (1990). Serum-dependent interaction of granulocytes with *Onchocerca volvulus *microfilariae in generalized and chronic hyper-reactive onchocerciasis and its modulation by diethylcarbamazine. Trans R Soc Trop Med Hyg.

[B12] Clancy RM, Abramson SB (1995). Nitric oxide: a novel mediator of inflammation. Proc Soc Exp Biol Med.

[B13] Sano H, Hla T, Maier JAM, Crofford LJ, Case JP, Maciag T, Wilder RL (1992). In vivo cyclooxygenase expression in synovial tissues of patients with rheumatoid arthritis and osteoarthritis and rats with adjuvant and streptococcal cell wall arthritis. J Clin Invest.

[B14] Salvemini D (1997). Regulation of cyclooxygenase enzymes by nitric oxide. Cell Mol Life Sci.

[B15] Clancy R, Varenika B, Huang W, Ballou L, Attur M, Amin AR, Abramson SB (2000). Nitric oxide synthase/COX cross-talk: nitric oxide activates COX-1 but inhibits COX-2-derived prostaglandin production. J Immunol.

[B16] Horii Y, Aoki Y (1997). Plasma levels of diethylcarbamazine and their effects on implanted microfilariae of *Brugia pahangi *in rats. J Vet Med Sci.

[B17] Denham DA, Suswillo RR, Rogers R, McGreevy PB (1978). Studies with *Brugia pahangi *17. The anthelmintic effects of diethylcarbamazine. J Parasitol.

[B18] Neill M, Kazura JW (1979). The effect of diethylcarbamazine in a murine model of *Brugia malayi *microfilaraemia. Bull WHO.

[B19] Taylor MJ, Cross HF, Mohammed AA, Trees AJ, Bianco AE (1996). Susceptibility of *Brugia malayi *and *Onchocerca lienalis *microfilariae to nitric oxide and hydrogen peroxide in cell-free culture and from IFNτ-activated macrophages. Parasitol.

[B20] Rajan TV, Shultz LD, Babu S, Doukas J, Greiner D, Porte P (1998). Diethylcarbamazine (DEC) does not induce nitric oxide (NO) synthesis. Exp Parasitol.

[B21] Salvemini D, Misko TP, Masferrer JL, Seibert K, Currie MG, Needleman P (1993). Nitric oxide activates cyclooxygenase enzymes. Proc Natl Acad Sci USA.

[B22] Salvemini D, Seibert K, Masferrer JL, Misko TP, Currie MG, Needleman P (1994). Endogenous nitric oxide enhances prostaglandin production in a model of renal inflammation. J Clin Invest.

[B23] Salvemini D, Settle SL, Masferrer JL, Seibert K, Currie MG, Needleman P (1995). Regulation of prostaglandin production by nitric oxide; an *in vivo *analysis. Br J Pharmacol.

[B24] Devaux Y, Seguin C, Grosjean S, de Talancé N, Camaeti V, Burlet A, Zannad F, Meistelman C, Moertes P-M, Longrois D (2001). Lipopolysaccharide-induced increase of prostaglandin E2 is mediated by inducible nitric oxide synthase activation of the constitutive cyclooxygenase and induction of membrane-associated prostaglandin E synthase. J Immunol.

[B25] Marnett LJ, Wright TL, Crews BC, Tannenbaum SR, Morrow JD (2000). Regulation of prostaglandin biosynthesis by nitric oxide is revealed by targeted deletion of inducible nitric-oxide synthase. J Biol Chem.

[B26] Swierkosz TA, Mitchell JA, Warner TD, Botting RM, Vane JR (1995). Co-induction of nitric oxide synthase and cyclo-oxygenase: interactions between nitric oxide and prostanoids. Br J Pharmacol.

[B27] Reddy ST, Tiano HF, Langenbach R, Morham SG, Herschman HR (1999). Genetic evidence for distinct roles of COX-1 and COX-2 in the immediate and delayed phases of prostaglandin synthesis in mast cells. Biochem Biophys Res Comm.

[B28] Bozza PT, Payne JL, Morham SG, Langenbach R, Smithies O, Weller PF (1996). Leukocyte lipid body formation and eicosanoid generation: cyclooxygenase-independent inhibition by aspirin. Proc Natl Acad Sci USA.

[B29] Hobbs MR, Udhayakumar V, Levesque MC, Booth J, Roberts JM, Tkachuk AN, Pole A, Coon H, Kariuki S, Nahlen BL, Mwaikambo ED, Lal AL, Granger DL, Anstey NM, Weinberg JB (2002). A new NOS2 promoter polymorphism associated with increased nitric oxide production and protection from severe malaria in Tanzanian and Kenyan children. Lancet.

[B30] Kun JF, Mordmuller B, Perkins DJ, May J, Mercereau-Puijalon O, Alpers M, Weinberg JB, Kremsner PG (2001). Nitric oxide synthase 2 (Lambarene) (G-954C), increased nitric oxide production, and protection against malaria. J Inf Dis.

[B31] Ohashi J, Naka I, Patarapotikul J, Hananantachai H, Looareesuwan S, Tokunaga K (2002). Significant association of longer forms of CCTTT microsatellite repeat in the inducible nitric oxide synthase promoter with severe malaria in Thailand. J Inf Dis.

[B32] Rutherford S, Johnson MP, Curtain RP, Griffiths LR (2001). Chromosome 17 and the inducible nitric oxide synthase gene in human essential hypertension. Hum Genet.

[B33] Cesbron JY, Capron A, Vargaftig BB, Lagarde M, Pincemail J, Braquet P, Taelman H, Joseph M (1987). Platelets mediate the action of diethylcarbamazine on microfilariae. Nature.

[B34] Chen LY, Mehta JL (1996). Further evidence of the presence of constitutive and inducible nitric oxide synthase isoforms in human platelets. J Cardiovasc Pharmacol.

[B35] Mehta JL, Chen LY, Kone BC, Mehta P, Turner P (1995). Identification of constitutive and inducible forms of nitric oxide synthase in human platelets. J Lab Clin Med.

[B36] Liu LX, Serhan CN, Weller PF (1990). Intravascular filarial parasites elaborate cyclooxygenase-derived eicosanoids. J Exp Med.

[B37] Liu LX, Buhlmann JE, Weller PF (1992). Release of prostaglandin E_2 _by microfilariae of *Wuchereria bancrofti *and *Brugia malayi*. Am J Trop Med Hyg.

[B38] Kaiser L, Lamb VL, Tithof PK, Gage DA, Chamberlin BA, Watson JT, Williams JF (1992). *Dirofilaria immitis *: do filarial cyclooxygenase products depress endothelium-dependent relaxation in the *in vitro *rat aorta?. Exp Parasitol.

[B39] Sommer A, Rickert R, Fischer P, Steinhart H, Walter RD, Liebau E (2003). A dominant role for extracellular glutathione S-transferase from *Onchocerca volvulus *is the production of Prostaglandin D_2_. Infect Immun.

[B40] Liu LX, Weller PF (1992). Intravascular filarial parasites inhibit platelet aggregation. Role of parasite-derived prostanoids. J Clin Invest.

[B41] Liu LX, Weller PF (1990). Arachidonic acid metabolism in filarial parasites. Exp Parasitol.

[B42] Peixoto CA, Rocha A, Aguiar-Santos A (2004). The effects of diethylcarbamazine on the ultrastructure of microfilariae of *Wuchereria bancrofti *in vivo and in vitro. Parasitol Res.

